# What is the impact of systems of care for heart failure on patients diagnosed with heart failure: a systematic review

**DOI:** 10.1186/s12872-016-0371-7

**Published:** 2016-10-11

**Authors:** Andrea Driscoll, Sharon Meagher, Rhoda Kennedy, Melanie Hay, Jayant Banerji, Donald Campbell, Nicholas Cox, Debra Gascard, David Hare, Karen Page, Voltaire Nadurata, Rhonda Sanders, Harry Patsamanis

**Affiliations:** 1 Deakin University, Locked Bag 20000, Geelong, VIC 3220 Australia; 2 Heart Foundation (Victoria), Level 12, 500 Collins st, Melbourne, 3000 Australia; 3 School of Rural Health, Monash University, Bendigo, Victoria Australia; 4 Monash Health, Clayton, Melbourne, Australia; 5 Cardiology Department, Western Health, Gordon Street, Footscray, 3011 Melbourne, Australia; 6 Monash Health, Monash Health Community, Dandenong, Melbourne, Australia; 7 Department of Cardiology, University of Melbourne and Austin Health, Burgundy St Heidelberg, 3081 Melbourne, Australia; 8 Cardiology Department, Bendigo Health, Victoria, Australia; 9 St Vincent’s Hospital, Victoria parade, Melbourne, Australia

**Keywords:** Heart failure, Systems of care, Hospital readmissions, Primary care, Hospitalisations, Transitional care, Workforce, Systematic review

## Abstract

**Background:**

Hospital admissions for heart failure are predicted to rise substantially over the next decade placing increasing pressure on the health care system. There is an urgent need to redesign systems of care for heart failure to improve evidence-based practice and create seamless transitions through the continuum of care. The aim of the review was to examine systems of care for heart failure that reduce hospital readmissions and/or mortality.

**Method:**

Electronic databases searched were: Ovid MEDLINE, EMBASE, CINAHL, grey literature, reviewed bibliographies and Cochrane Central Register of Controlled Trials for randomised controlled trials, non-randomised trials and cohort studies from 1^st^ January 2008 to 4^th^ August 2015. Inclusion criteria for studies were: English language, randomised controlled trials, non-randomised trials and cohort studies of systems of care for patients diagnosed with heart failure and aimed at reducing hospital readmissions and/or mortality.

Three reviewer authors independently assessed articles for eligibility based on title and abstract and then full-text. Quality of evidence was assessed using Newcastle-Ottawa Scale for non-randomised trials and GRADE rating tool for randomised controlled trials.

**Results:**

We included 29 articles reporting on systems of care in the workforce, primary care, in-hospital, transitional care, outpatients and telemonitoring. Several studies found that access to a specialist heart failure team/service reduced hospital readmissions and mortality. In primary care, a collaborative model of care where the primary physician shared the care with a cardiologist, improved patient outcomes compared to a primary physician only. During hospitalisation, quality improvement programs improved the quality of inpatient care resulting in reduced hospital readmissions and mortality. In the transitional care phase, heart failure programs, nurse-led clinics, and early outpatient follow-up reduced hospital readmissions. There was a lack of evidence as to the efficacy of telemonitoring with many studies finding conflicting evidence.

**Conclusion:**

Redesigning systems of care aimed at improving the translation of evidence into clinical practice and transitional care can potentially improve patient outcomes in a cohort of patients known for high readmission rates and mortality.

## Background

Approximately 1–3 % of the adult population have been diagnosed with heart and one in five people will develop heart failure during their lifetime with the incidence increasing with age [1]. The prognosis of heart failure is poor with a 10 % in-hospital mortality rate from acute heart failure, post-discharge 20–40 % mortality rate within one year, and 20–25 % will be readmitted within one month [2–7]. Over the natural course of heart failure, people will experience acute episodes requiring urgent medical treatment and hospitalisation. Hospital admissions for heart failure are predicted to rise substantially over the next decade placing increasing pressure on the health care system as health care costs associated with heart failure will also dramatically rise. There is an urgent need to redesign health systems of care for heart failure to improve evidence-based practice and create seamless systems of care across the health care continuum embracing primary care, hospital and community care. This literature review will systematically review articles that focus on systems of care for heart failure aimed at reducing hospital readmission and mortality rates. A system of care is defined as one or several interventions implemented for service delivery in health care.

## Methods

### Types of studies

Studies included in the review implemented an intervention or interventions involving health service delivery aimed at reducing hospital readmissions and mortality for patients diagnosed with heart failure. Patients diagnosed with heart failure with reduced (HFrEF) and/or preserved ejection fraction (HFpEF) were included. All of the studies included in the review had received ethics approval.

### Search methods

The search strategy was based on the PRISMA statement [8]. The following databases were searched for studies of systems of care for heart failure:

1. The Cochrane Central Register of Controlled Trials (CENTRAL, issue 7 of 12, searched 4/8/2015, results: 9)
2. MEDLINE (EBSCO host, 2008 to August week 1 2015, searched 4/8/2015, results: 145)
3. EMBASE (EMBASE platform, 2008 to 2015 week 31, searched 4/8/2015, results: 107)
4. CINAHL (EBSCO host, 2008 to August week 1 2015, searched 4/8/2015, results: 21)

The Cochrane Highly Sensitive Search Strategy was used for MEDLINE and an adaptation of it for EMBASE and CINHL [9]. See Appendix 1 for details of the search strategies. We restricted the search to studies reported in English and from 1^st^ January 2008 to 4^th^ August 2015. There was no restriction on study design. We included randomised controlled trials, non-randomised trails and observational studies. All citations were imported into EndNote XVII^TM^ electronic database.

The following clinical trials registries were also searched: WHO International Clinical Trial Registry Platform (ICTRP) (www.who.int/trialsearch) and clinical trials (www.clinicaltrial.gov ) (searched 5^th^ August 2015). Full reference lists of key eligible papers and review articles were searched to identify potential papers. We also searched the grey literature to identify unpublished theses, policy documents and abstracts. Reference lists of heart failure guidelines (national and international) and other systematic reviews and meta-analyses were also searched.

#### Selection of studies

All titles and abstracts were assessed for eligibility by three authors working independently. If the title and abstract contained sufficient information to determine exclusion, it was rejected. Where the type of intervention or study population was not clear from the title or abstract the full text of the paper was retrieved and evaluated to determine inclusion or exclusion. The reference lists of eligible papers were reviewed to identify potential papers. The principle reason for exclusion of papers and abstracts was documented based on inclusion criteria. Disagreements between the reviewers were resolved by discussion and consensus between the three authors.

#### Assessment of quality of evidence for non-randomised controlled trials

The Newcastle-Ottawa Scale (NOS) was used to assess the quality of non-randomised studies [10] (Table 1). This tool has been used previously in Cochrane Reviews for assessment of risk of bias in non-randomised studies with high inter-rater reliability and content validity [10]. The NOS comprises of eight items: representativeness of cohort, selection of cohort, ascertainment of exposure, outcome of interest was not present at baseline, comparability of cohorts, assessment of outcome, length of follow-up and adequacy of follow-up. When the paper under review met the criterion in the NOS, it was awarded a ‘*’. A paper was also awarded an additional ‘*’ if the analysis was adjusted for potential confounding variables. The quality of each study was graded as low, medium or high according to the number of stars (*).

**Table 1:** Summary of Quality Assessment (Newcastle-Ottawa Scale): Non randomised studies

Study	Selection				Comparability of cohorts^a^	Outcome			Evidence quality^b^
	Exposed cohort representative	Non exposed cohort selection	Exposure ascertainment	Outcome not present at start		Assessment	Follow-up length	Follow up adequacy	
Workforce									
Zuily, 2010 [15]	*	*	*	*	**	*	*	*	High
Boom, 2012 [13]	*	*	*	*	**	*	*	*	High
NICOR, 2012 [12]	*	*	*	*	**	*	*	*	High
Comin-Colet, 2014 [16]	*	*	*	*	**	*	--	--	High
Primary care									
Lee, 2010 [18]	*	*	*	*	**	*	*	*	High
Rosstad, 2013 [17]	*	*	*	*	--	--	--	--	Low
In-hospital studies									
Williams, 2010 [21]	*	*	*	*	--	*	*	*	Low
Tuso, 2014 [22]	*	*	*	*	--	*	*	*	Low
In-hospital clinical audits/registries/quality improvement initiatives									
Boutwell, 2011 [33]	*	*	*	*	--	NA	NA	NA	Low
Heidenreich 2012 [26]	*	*	*	*	**	*	*	*	High
Hansen, 2013 [32]	*	*	*	*	*	*	*	*	Moderate
H2H National Quality Improvement Initiative, 2015 (H2H program) [34]	*	*	*	*	--	NA	NA	NA	Low
Transitional care									
Driscoll, 2011 [45]	*	*	*	*	**	*	*	*	High
Outpatient clinics									
Fonarow, 2011 [51]	*	*	*	*	**	*	*	*	High
Hernandez, 2010 [49]	*	*	*	*	**	*	*	*	High
Fenner, 2014 [50]	*	*	*	*	--	-	*	*	Low
Telemonitoring programs									
Piette, 2008 [60]	*	*	*	*	--	-	*	*	Low
Baker, 2013 [61]	*	*	*	*	**	*	*	*	High

*NA* not applicable as outcome data has not been reported at the time of the literature search

^a^Also includes controlling for potential confounders

^b^Evidence quality

Low: downgrading from moderate to low based on design or lack of information in report

Moderate: study met selection criteria (4 stars), comparability (1 star and upgraded a level for 2 stars), and outcome assessment

High: upgrading from moderate to high based on comparability of 2 stars

#### Assessment of risk of bias for randomised controlled trials

The Cochrane Collaboration tool for risk assessment [11] was used. Each randomised controlled trial was assessed for selection bias, performance bias, attrition bias, and detection bias (Table 2). The risk of bias was assessed as low, high or unclear. Study quality was not a reason for exclusion of a study.

**Table 2:** Risk of bias: Randomised controlled trials

Author, year	Random sequence generation	Allocation concealment	Blinding of participants and personnel	Blinding of outcome assessment	Incomplete outcome data	Selective reporting	Other bias	Overall risk of bias
Transitional care7								
Jaarsma, 2008 [44]	Low	Unclear	High	Low	Low	Low	None	Low
Nurse-led outpatient clinic								
Driscoll, 2014 [45]	Low	Unclear	High	Unclear	Low	Low	None	Low
Telemonitoring programs								
Schwarz, 2008 [59]	Unclear	Low	High	Unclear	Unclear	Low	None	Unclear
Woodend, 2008 [57]	Unclear	Low	High	Unclear	Low	Low	None	Low
Chaudhry, 2010 [55]	Low	Unclear	High	Low	Unclear	Low	None	Low
Koehler, 2011 [52]	Low	Unclear	High	Low	Low	Low	None	Low
Angermann, 2012 [39]	Low	Low	High	Low	Low	Low	None	Low
Dendale, 2012 [54]	Low	Low	High	Low	Low	Low	None	Low
Pekmezaris, 2012 [56]	Low	Unclear	High	Unclear	Low	Low	None	Low
Krum, 2013 [53]	Low	Low	Low	Low	Low	Low	None	Low
Black, 2014 [58]	Low	Low	Low	Unclear	Unclear	Unclear	None	Unclear

### Data synthesis

Due to the diversity associated with the design and outcome measures in observational and cohort studies, meta-analysis for pooled estimates was not conducted so the data was synthesised qualitatively and consisted of a narrative synthesis of the evidence.

## Results

A total of 520 studies were identified from the literature search. After removing the duplicate articles we reviewed the titles and abstracts of 487 articles. Of these abstracts, 212 full-text articles were identified for retrieval and possible inclusion in the literature review. From the full-text articles we excluded 183 studies. We included 29 studies in the integrated literature review.

**Fig. 1:**
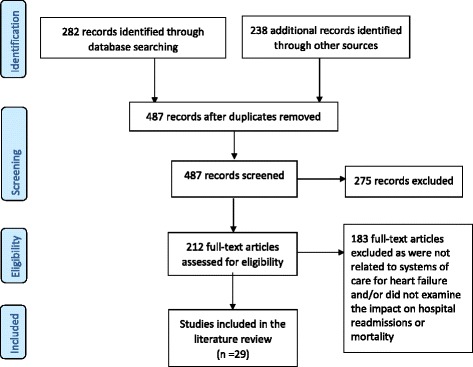
Study flow diagram

The PRISMA flow diagram in Fig. 1 outlines the selection process of studies included in the literature review. The studies were classified into four main categories, based primarily on the environment of delivery, relating to systems of care for heart failure: workforce, primary care, in-hospital and transitional/community systems of care. Workforce was included as a category as often the intervention spanned across multiple environments such as primary care, in-hospital and community. Appendix 2 provides a summary of the included studies. Systematic reviews, meta-analyses and policy documents have been excluded from Appendix 2.

### Specialist workforce

When redesigning systems of care, an experienced workforce is critical to its success. Three studies examined the impact of workforce on patient outcomes. All of these studies were rated as a high quality of evidence (Table 2). Specialist heart failure teams within the community and in-hospital were associated with improved patient outcomes. A UK national heart failure audit, conducted between April 2010 to March 2011, found in-patient mortality rates were better in patients admitted under Cardiology (8 %) compared to General Medicine (14 %) and other wards (17 %) [12]. In another study, these benefits were also extended to lower 30-day and 12 month mortality rates [13]. Boom and colleagues [13] recruited 7634 patients newly hospitalised with heart failure. Patients who were admitted under a generalist physician and had a cardiologist involved in their care were more likely to undergo cardiac investigations during their inpatient stay. Patients treated by a generalist physician only were also at increased risk of 30-day mortality (odds ratio [OR] 1.50, 95 % confidence interval [CI] 1.18–1.91) compared to patients that were admitted under a generalist physician and had a cardiologist involved in their care [13].

Improved outcomes were also seen in outpatient settings with lower mortality rates for patients followed up with cardiology clinics (18 %) compared to non-cardiology clinics (31 %) [12]. There was also a similar trend with patients followed up with heart failure specialist nursing services (22 % mortality rate) compared to follow up with a non-heart failure specialist nurse (27 %) [12]. A dedicated in-hospital heart failure unit also showed further reduction in heart failure readmissions and all-cause event-free survival [14, 15].

Comin-Colet and colleagues [16] implemented a health service-wide heart failure program encompassing an inpatient service, community service and a heart failure unit including a multidisciplinary specialist heart failure team. They examined hospital readmissions and mortality rates of 2083 patients, admitted with acute decompensated heart failure, to the hospital with a heart failure service compared to the outcomes associated with 54 659 patients admitted, with acute decompensated heart failure, to hospitals with no heart failure service within the surrounding region. They found that patients admitted to the hospital with the heart failure service had a lower risk of death (hazard ratio [HR] 0.92, 95 % CI 0.86–0.97), 29 % less likely to experience a readmission for any cause (HR 0.71, 95 % CI 0.66–0.76), and 14 % lower risk of heart failure readmissions (HR 0.86, 95 % CI 0.80–0.94) [16].

### Primary care

Most of the literature examining systems of care for heart failure in primary care focused on a collaborative model of care and adherence to clinical guidelines. Of the two studies included, one was rated as a low [17] quality of evidence and the other as high [18] (Table 1). Lee et al. [18] examined readmission rates of 10 599 heart failure patients discharged from emergency department (ED) who were managed in a collaborative care model comprising of a cardiologist and general practitioner compared to general practitioner only or no follow-up. Collaborative care (cardiologist and general practitioner) reduced mortality compared with general practitioner only (HR 0.79; 95 % CI 0.63 to 1.00) [18].

Care pathways have also been trialed in primary care. Roostad [17] found that a disease-based care pathway was ineffective and unsustainable in primary care. This was mainly due to multiple co-morbidities that can be associated with heart failure and the need for multiple care pathways for each co-morbidity which may be contradictory to other care pathways. This study was rated as low quality of evidence as there was no follow up.

### In-hospital care

Many studies implemented interventions to improve the translation of clinical practice guidelines into standard patient care with the aim of reducing 30 day readmissions. Hansen [19] undertook a systematic review of interventions implemented pre and post discharge to reduce 30-day readmissions. Pre-discharge interventions included: patient education, medication reconciliation, discharge planning, and scheduling of a follow-up appointment before discharge. Post-discharge interventions comprised of: follow-up telephone calls, patient activated hotlines, timely communication with ambulatory providers, timely ambulatory provider follow-up, and post-discharge home visits. Bridging interventions included transition coaches, physician continuity across the inpatient and outpatient setting, and patient centered discharge instruction. Hansen [18] found that no single intervention alone was associated with reduced risk for 30-day readmissions rather bundles of interventions were more effective. Care pathways were also associated with improved patient outcomes.

In the acute hospital setting, a meta-analysis of care pathways reported reductions in readmission (RR 0.81, 95 % CI 0.66–0.99) and in-hospital death rates (RR 0.45, 95 % CI 0.21–0.94) compared with usual care in patients hospitalized with acute heart failure [20]. However, the meta-analysis combined results from three randomised controlled trials, one interrupted- time series and three controlled trials so differences in patient characteristics may have affected the outcomes [20]. A limitation of care pathways is that one standardised care pathway will not be suitable for all hospitals so effectiveness varies greatly and the results of the meta-analysis should be interpreted with caution. Care pathways were also not found to be effective or sustainable in primary care [17].

In addition to a systematic review and meta-analysis, two studies implemented a system of care during the inpatient phase. Williams [21] implemented a quasi‑experimental study to determine the effectiveness of an inpatient education program and discharge planning on reducing hospital readmission. All patients were followed up for 18 weeks post-discharge. They found no difference between groups for 30 day hospital readmissions. There was a significantly shorter length of hospital stay for patients in the education program compared to no education (10.68 days versus 9.58 days, *p* = 0.05) [21]. The study had a low quality of evidence due to lack of controlling for confounding variables (Table 1).

One study implemented a ‘heart failure bundle’ [22]. This study was rated as a low quality of evidence mainly due to a lack of controlling for confounding variables during data analysis (Table 2). The bundle included: inpatient heart failure education, a home visit within 48 h of discharge, and a follow-up appointment and follow-up phone call from a heart failure care manager both within seven days of discharge. Readmissions rates were reduced from 19 to 15 % over 30-days (*p* = 0.03) [22].

### In-hospital quality of care performance initiatives

Several studies discussed the implementation of large quality improvement programs and clinical inpatient registries to improve hospital management of heart failure patients. Considerable variation in the management of heart failure between hospitals exists [12, 21, 23]. Several studies showed an improvement in survival and reduction in readmission rates as patients with heart failure were cared for in hospitals that complied with clinical practice guidelines compared with hospitals with low compliance rates [23–26].

#### Clinical audits/registries

There are several national registries throughout the world: HEARTS in Saudi Arabia, CHART-2 in Japan, GULF CARE in Middle East, and ASIAN-HEART FAILURE in Asia [27]. Results from these registries are yet to be published. The implementation of many large clinical registries and clinical audits occurred prior to the time period of this literature search such as OPTIMISE [24], and ADHERE [23] and EuroHeart failure survey I [28] and II [2]. However, one large clinical audit was the UK national audit [12]. This study was assessed as a high quality of evidence rating (Table 2).

##### UK national heart failure audit

In England and Wales, over the past four years, an annual national audit of patients admitted to hospital with acute decompensated heart failure has been undertaken. The latest national audit conducted between April 2010 and March 2011 collected data on 36 items (based on national guidelines) from 133 National Health Service Trusts and Welsh Health Boards on 36 504 patients representing 54 % of all hospital admissions for acute decompensated heart failure [12]. The audit found a large degree of heterogeneity in the management of heart failure across hospitals particularly the proportions of patients undergoing key diagnostic tests, receiving cardiovascular medications on discharge and being referred to cardiology follow-up services [12]. In-hospital mortality was lower for those patients admitted under Cardiology (8 %) compared to those patients admitted under General Medicine (14 %) and other Units (17 %). This trend also extended to post-discharge with an 18 % mortality rate with Cardiology follow-up compared to 31 % with non-cardiology follow up [12]. A similar trend was seen with patients followed by a specialist heart failure nurse at 22 % mortality compared to 27 % with no follow up with a heart failure specialist nurse. Unfortunately, hospital readmissions were not collected in the audit [12].

#### Quality improvement initiatives

Several nationwide quality improvement initiatives have also been implemented with the aim of reducing 30-day hospital readmissions. These include: Get with the Guidelines (GWTG)-HEART FAILURE, Better Outcomes for Older adults through safe transitions (BOOST) project, State Action on Avoidable Rehospitalisations (STAAR) program, and Hospital-to-Home program (H2H).

##### GWTG-heart failure

The GWTG program was implemented by American Heart Association to address the gap in implementation of evidenced-based clinical practice guidelines with the aim of improving patient outcomes post discharge. Several disease-specific GWTG programs have been implemented: GWTG-Atrial Fibrillation, GWTG-Resuscitation, and GWTG-stroke. Registered hospitals receive access to GWTG toolkit specific for heart failure. The toolkit comprises of: initiation of evidence-based medications, implantation of appropriate device therapies, discharge education about heart failure, evaluation of left ventricular function, and post discharge follow-up appointment [29]. Data is then entered into a web based system and each participating hospital receives site level patient data to identify those at risk of readmission. The program also provides professional education, patient education resources, and clinical support tools. GWTG currently has data from over 5 million patients and over 2093 US hospitals that participated at least once in GWTG (http://www.heart.org/HEARTORG/HealthcareResearch/GetWithTheGuidelinesHEART FAILUREStroke/GetWithTheGuidelinesHeartFailureHomePage/Get-With-The-Guidelines-Heart-Failure-Home-Page_UCM_306087_SubHomePage.jsp) [29]. Results have shown an improvement in adherence to performance measures and systems of care but subsequent impact on patient outcomes has been disappointing [26, 30, 31]. Readmissions at 30-days was 24.5 % and mortality at 30 days was 11 % [26]. Readmission rates [26] and mortality [31] were significantly lower in hospitals participating in GWTG- heart failure. This study [26] had a high quality of evidence (Table 1).

##### BOOST project

(Better Outcomes for Older adults through safe transitions) [32]. This was a quality improvement project to reduce hospital readmissions and length of stay for patients hospitalised with heart failure. Thirty hospitals enrolled in the project but only eleven submitted their data. In 2014, 180 hospitals were involved in the project [32]. The BOOST intervention consisted of a toolkit which contained: an implementation guide, project management tools, such as the Teach Back Training Curriculum, and PICO guidelines to evaluate the intervention. Sites were also provided with face‐to‐face training and 12 months of expert mentoring and coaching, and assistance to build a culture that supports organisational change to reduce hospital readmissions, also linking with other participating sites and data management. Each site also received project benchmark data and site level patient data. The average rate of 30-day rehospitalisation was 15 % pre-implementation and 13 % 12 months post-implementation [32]. There was no difference in length of stay. Unfortunately, not all of the hospitals implemented all of the quality tools, with the majority implementing two of the five BOOST tools. This study was rated as a moderate quality of evidence due to not adjusting for confounding variables (Table 1).

#### STAAR program

State Action on Avoidable Rehospitalisations initiative [33]. This program involved a state- based approach to reducing 30-day hospital readmissions. It involved 148 hospitals partnering with community based organisations with the aim of improving communication and the transition between health care providers. The intervention also comprised of multi-stakeholder, state-level steering committees. These committees coordinated programs throughout the State aligning partners and troubleshooting barriers to implementation [33]. The collaborative cross-continuum teams included health professionals from acute care, community health programs, Aged Care facilities, ambulatory care, social services, patient and family caregivers. Each hospital was expected to perform a comprehensive assessment of patients’ needs post-discharge, provide patient and carer education, provide clear discharge information to the patient, carer and community provider and ensure early post-discharge follow-up for medical and non-medical services. At the time of writing the systematic review, results from STAAR were not published. The study was rated as a low quality of evidence due to a lack of information about their outcome data.

##### Hospital to home (H2H)

The Hospital to Home (H2H) [34] Initiative provided a toolkit to clinicians to assist them in implementing evidence-based care from clinical guidelines, within their organisation. The toolkit contained evidence-based clinical information, webinars and recommended strategies and tools to improve evidence-based practice. The goal of the toolkit was for all patients to have a follow-up appointment/cardiac rehab referral within seven days of discharge, improved medication management, and information about early warning signs and a care plan to address them [34]. At the time of the conducting this literature search the only published article was an evaluation done by Bradley [35].

Bradley [35], undertook a survey of 537 hospitals enrolled in the H2H program, to determine their resources for reducing heart failure readmissions. All of the hospitals reported monitoring their performance data in particular 30-day readmissions rates. Two thirds of the hospitals had a designated person or group to review unplanned readmissions that occurred within 30 days of discharge. On average, hospitals implemented less than half of the 10 recommended H2H practices. Less than 3 % of the hospitals, routinely used all 10 [35]. The H2H programs was rated as low quality of evidence due to a lack of published information about their outcome data (Table 1).

### Transitional/community based care

Most transitional care literature focussed on post-discharge heart failure programs and implementing single site specific interventions. Numerous meta-analyses have shown that heart failure programs reduce hospital readmissions and mortality [36–38]. Meta-analyses of heart failure programs found a large degree of heterogeneity between studies and no single intervention was able to be isolated to determine their effectiveness. Rather the effectiveness of many discharge programs was due to a bundle of interventions [16, 22, 40, 41]. A quality improvement tool was developed from a national survey of heart failure programs and data from 573 patients enrolled in those programs [40]. The quality improvement tool showed that the more interventions implemented within a program the greater the improvement in patient outcomes. Patients participating in complex programs were 20 % less likely to experience a hospital readmission and/or mortality (HR 0.80, 95 % CI 0.70–0.92) compared to less complex programs [40]. This study was rated with a high quality of evidence (Table 1).

The use of specialist heart failure nurses within the heart failure programs also improved patient outcomes [12]. Several meta-analyses of heart failure programs have shown an improvement in patient outcomes in programs where nurses are experienced in heart failure and have qualifications in a cardiac speciality and/or critical care. A randomised control trial of a heart failure program involving generic nurses with no cardiac experience found a 13 % reduction in hospital readmissions [42] compared to meta-analyses involving heart failure nurses showing a 30 % reduction in hospital readmissions [36, 43].

Jaarsma and colleagues [44] implemented a randomised controlled trial to determine the effect of low, moderate or high intensity, post-discharge follow up with a heart failure nurse. Low intensity follow-up or usual care comprised of an outpatient appointment with a Cardiologist within two months post-discharge and then every six monthly. Moderate follow-up consisted of usual care and nine outpatient appointments with a heart failure nurse. High intensity follow-up also consisted of usual care and weekly telephone calls and a home visit within the first month post-discharge, followed by additional telephone calls with the heart failure nurse, two home visits and two multidisciplinary appointments. They found that neither moderate nor intensive follow up by a heart failure nurse reduced the combined end points of heart failure death and hospitalization compared with usual care. At 18 months, 411 patients (40 %) were readmitted because of heart failure or died from any cause [44]. There was no significant difference in heart failure readmission or mortality between the three groups: 42 % in the control group, and 41 % and 38 % in the basic and intensive support groups, respectively (*P* = .73 and *P* = .52, respectively) [44].

### Nurse-led medication titration

Nurse-led medication titration in heart failure patients has been shown to improve patient outcomes whether in a clinic or in the community. Driscoll and colleagues [45] examined nurse-led titration of beta-adrenergic blockers by heart failure nurses in the community during a home visit. They recruited 484 patients diagnosed with HFrEF participating in 33 heart failure programs. The study found all-cause hospitalisations and mortality was lower in patients participating in programs allowing nurse-led titration of beta-adrenergic blocking agents (HR 0.58, 95 % CI 0.42–0.81) [45]. The study was assessed as a high quality of evidence (Table 1). Driscoll et al. [46] also implemented a randomised controlled trial of a nurse-led titration clinic for patients diagnosed with HFrEF. Patients were randomised to titration of beta-adrenergic blocking agents by a nurse in an outpatient clinic or follow up by their general practitioner for titration of these medications [46]. The nurse-led medication titration clinic resulted in a 50 % reduction in time to optimal dose of beta-adrenergic blocking agents compared with optimisation of beta-adrenergic blocking agents by general practitioners (90 ± 14 days vs 166 ± 8 days, *p* < 0.0005) [46]. Risk of bias was assessed as low (Table 2). A meta-analysis of nurse-led titration, regardless of the setting, found that patients participating in nurse-led titration of beta-adrenergic blocking agents and angiotensin converting enzyme inhibitors were 21 % less likely to be readmitted for any cause (HR 0.79, 95 % CI 0.36–0.72) and 34 % were less likely to die [47].

### Outpatient clinics

Literature involving outpatient clinics was mainly concerned with a lack of follow-up post-discharge and the benefits of follow-up in a heart failure clinic compared to generalist clinics. A recent analysis of Medicare claims data in the USA, found that of the patients hospitalised for heart failure, 52 % of patients did not have an outpatient visit [48].

Heart failure clinical guidelines recommend early follow-up within 7–10 days post-discharge [49–51]. GWTG-HEART FAILURE found the median percentage of patients who had early follow-up after discharge from the index hospitalization was 38.3 % (interquartile range, 32.4 %–44.5 %) [49]. There was a large degree of variation between hospitals for early outpatient follow-up after discharge. Patients who had higher early follow-up appointments had a lower risk of 30-day readmission [49]. This study was rated as a high quality of evidence (Table 2). Another study implemented follow-up at a heart failure clinic, within three days post-discharge [50]. There was a reduction in heart failure readmission rate from 18 % to 13 % [50]. The heart failure service was then extended to include telemedicine, using basic videoconferencing for patients living in rural and remote areas. Heart failure readmissions for this group of patients was reduced from 18 % to 10 % over a six month period [50]. There was a low quality of evidence for this study mainly due to no adjustment for potential confounding variables (Table 1).

The Registry to Improve the Use of Evidence-Based Heart Failure Therapies in the Outpatient Setting (IMPROVE-HF) is a national registry and performance improvement program of 15381 patients with chronic HFrEF from 167 outpatient cardiology practices [51]. The IMPROVE-HF study found an increase in adherence in performance measures was significantly associated with improved survival [51]. This study was rated as a high quality of evidence (Table 1).

### Telemonitoring/telehealth

Telemonitoring is another transitional care intervention, particularly for those patients who do not have access to a Cardiologist or heart failure nurse. Telemonitoring involves automated transmission of patient data to a central service and includes measures such as patient-measured weight, blood pressure, heart rate and heart rhythm. A study by Cleland and colleagues [6] found mortality rates at 12 months were lower in patients participating in telemonitoring (29 %) or regular telephone support from a nurse (27 %) compared with usual care (45 %). In contrast, Koehler and colleagues [52] found no differences in mortality between the telemonitoring and usual care groups over 12–28 months. Nine randomised controlled trials investigated the effect of telemonitoring on hospital readmission and/or mortality. The risk of bias associated with these studies was low in seven studies [52, 39, 53–57] and unclear in two [58, 59] (Table 2). There were also two non-randomised studies that implemented a telemonitoring system, one study was rated as low [61] quality due to the lack of controlling for potential confounders and the other study was rated as high [61].

The Chronic Heart Failure Assessment by Telephone (CHAT) study investigated the utility of a telephone-based automated telemedicine system for patients diagnosed with HFrEF and living in rural and remote Australia [53]. The participants were required to dial into the telemedicine system monthly. The patients were required to answer questions about their heart failure clinical status, medical management of their condition and social questions relevant to their heart failure status. Alerts were set up within the Telewatch system alerting the CHAT nurse via the Patient Watch Screen to follow up patients that reported pre specified signs or symptoms warranting intervention. In patients randomised to the telemedicine system there were fewer patients hospitalised for any cause (74 versus 114, adjusted HR 0.67 [95 % CI 0.50–0.89], *p* = 0.006). Also less patients died and/or were hospitalised (89 versus 124, adjusted HR 0.70 [95 % CI 0.53–0.92], *p* = 0.011), compared to the usual care group [53]. The risk of bias associated with this study was low (Table 2).

The ‘telemonitoring in the management of heart failure’ (TEMA-HF) study did find a significant difference in mortality but not in readmission rates [54]. They randomised 160 patients hospitalised with heart failure to usual care or an intervention group. Patients randomised to usual care received a cardiology outpatient clinic within two weeks of discharge. Patients in the intervention group were provided with a telemonitoring system to use at home with in-built alerts when the patient’s vital signs fell below a predetermined level prompting follow up by a heart failure nurse, and a consultation service between the general practitioner and cardiologist concerning clinical management of the patient. At six months, all-cause mortality was significantly lower in the telemonitoring group compared to usual care (5 % versus 17.5 %, respectively, *p* = 0.01) but there was no significant difference in heart failure hospitalisations between the telemonitoring and usual care groups (0.24 versus 0.42 hospitalisations/patient, respectively, *p* = 0.06) [54].

The Health Buddy Program integrated a telehealth system with care management [61]. The program had 15 % lower risk-adjusted all-cause mortality (HR 0.85, 95 % CI 0.74–0.98; *P* = .03) and had reductions in the number of quarterly inpatient admissions from baseline to the study period that were 18 % greater than those of matched controls during this same time period [61]. There was a high quality of evidence associated with this study (Table 1). The BEAT-HF study is currently underway [58]. Patients will be randomised into an intensive patient education group using the ‘teach-back’ method and receive instruction in using the telemonitoring equipment. Following hospital discharge, they will receive a series of nine scheduled health coaching telephone calls over 6 months from nurses located in a centralized call center. The nurses will call patients and patients’ physicians in response to alerts generated by the telemonitoring system, based on predetermined parameters [58]. As results are currently not available the risk of bias associated with this study was unclear (Table 2).

A systematic review and meta-analysis of telemonitoring programs [63, 62] found a lower all-cause readmission rate and mortality for patients participating in a telemonitoring program. However, since that time, two large telemonitoring RCTs have found that telemonitoring had no effect on reducing hospital readmissions or mortality [52, 56]. Chaudhry [55] implemented an RCT of telemonitoring in 1653 patients recently admitted to hospital for acute decompensated heart failure. The telemonitoring program consisted of a telephone-based interactive voice response system that collected daily information about symptoms and weight that were reviewed by the patients’ clinicians. Patients were asked to dial into the system daily. During each call patients were asked several questions about their general health and heart failure symptoms. Chaudhry [55] found no difference in hospital readmission and mortality between the telemonitoring group and usual care. The study was reported as a low risk of bias (Table 2).

Koehler and colleagues [52] also implemented a telemonitoring program involving the randomisation of 710 chronic heart failure patients to a telemonitoring program or usual care. Similar to Chaudhry et al. [55], this study was also rated as a low risk of bias (Table 2). The telemonitoring program consisted of: portable devices for ECG, blood pressure, and body weight measurements. Patients were required to undertake daily self-assessments and the data was transferred to the telemonitoring data centre. They found no significant difference in all-cause mortality or heart failure hospitalisation [52]. Pekmezaris and colleagues [56] also implemented a similar telemonitoring system and randomised 168 patients, post hospitalisation for heart failure, into usual care or telemonitoring groups. They found no significant differences in 30 and 90-day readmission rates between usual care and telemonitoring groups [56]. Schwarz and colleagues [59] also randomised 102 patients and their carers to usual care or telemonitoring and found no significant differences between the groups for hospital readmissions at 90 days post-discharge (13 versus 12 respectively, *p* = 0.6) [60]. The risk of bias was assessed as unclear as only an abstract was available. Woodend and colleagues [57] also found a non-significant difference between their telemonitoring and usual care groups for hospital readmission at 90 days (5.48 % difference between groups, *p* > 0.05) and at one year post-discharge (−4.17 % difference, *p* > 0.05) [57].

Angermann and colleagues [39] also found no significant differences in their primary endpoint of hospital readmission rates, between usual care and telemonitoring groups. Angermann et al. [39] randomised 715 patients hospitalised with acute decompensated heart failure into one of two groups: usual care consisted of discharge planning and follow up with a cardiologist within 7–10 days post-discharge (363 patients) or to HeartNetCare-HF (HNC) comprising of inpatient visits with a heart failure nurse, structured telephone-based monitoring system including blood pressure, pulse and symptoms, uptitration of key medication and access to specialist care as required (352 patients) [39]. All patients were followed up for 180 days. There was no significant difference in the composite endpoint of all-cause hospitalisation or mortality (HR, 1.02; 95 % CI, 0.81, 1.30; *P* = 0.89) [39]. There was a slightly higher rate of readmissions in the HNC group compared to usual care (119 versus 112 respectively) but this was not statistically significant between the groups [39].

## Discussion

There was a paucity of studies that focussed on systems of care for heart failure with a primary outcome of readmission rates. Several studies recommended the implementation of a heart failure service or unit to manage heart failure patients regardless of the setting. Unfortunately, the translation of this evidence into clinical practice is poor, contributing to higher readmission and mortality rates [12, 64–66].

In primary care, several studies implemented a collaborative model of care for the management of patients diagnosed with heart failure. General practitioners are the cornerstone of managing heart failure patients in the community. However, there were only a few studies that were based in primary care. Difficulty in diagnosing heart failure due to the non-specific signs and symptoms can be challenging for general practitioners and may partly explain the under diagnosis of heart failure. Vilesca and colleagues [67] found that a general practitioner in primary care had a total of 10 guidelines that addressed diagnostics in heart failure. However, the criteria for diagnosis varied greatly [67]. This may contribute to a 12 % misdiagnosis rate for heart failure when general practitioners are responsible for the initial diagnosis [68]. The paucity of randomised controlled trials in primary care, focussing on improving systems of care for heart failure patients, highlights the need for more research in this area.

The main driver in improving inpatient systems of care for heart failure has been the 30-day readmission quality performance indicator in the USA. As a consequence, several large national quality improvement programs and clinical registries have been implemented throughout the USA. These programs have been effective in improving the translation of clinical guidelines into practice. However, reducing 30-day readmissions remains elusive. This raises the question: are 30 day readmissions the appropriate benchmark to use for reimbursement and as an indicator of hospital quality? Focussing on 30 days readmissions may under estimate the burden of heart failure and perhaps aiming our interventions at reducing heart failure readmissions over a longer period of time would be more effective. Over the last few years, hospitalisations, length of stay and in-hospital mortality for heart failure, have all improved, however, 30 day readmissions have not. It is unclear if this is due to inherent problems with the clinical indicator or inadequate progress with improving discharge planning and transitional care. Due to the chronicity associated with heart failure, effective systems of care need to encompass an outcome measure of readmission rates greater than 30 days. In order to improve hospital readmissions for heart failure a national co-ordinated approach is vital, with national benchmarking and collaboration between health professionals, particularly focusing on improving systems of care for managing inpatient heart failure patients.

A smooth, safe and efficient transition from hospital to home is essential to avoid hospital readmissions. The challenge is ensuring a seamless transition from hospital to outpatient care to long-term community care whilst not compromising on quality or adherence to evidence-based practice and maintaining linkage with a heart failure specialist team. Transitional care incorporates heart failure programs in the community and outpatient clinics. There was a high level of evidence supporting the implementation of nurse-led medication titration clinics. A meta-analysis of nurse-led clinics for the titration of key therapeutic medications reduced hospital readmissions and mortality [47]. An outpatient clinic appointment within 7–10 days post-discharge was also associated with a lower risk of hospital readmission [49]. In particular, an early follow-up with a cardiologist and their general practitioner improved patient survival [18]. Several clinical registries have also been implemented to monitor the quality of outpatient care. They all report an improvement in adherence to guideline recommended therapy [49, 51].

Several transitional programs also included telemontioring or telehealth. However, due to conflicting results between large randomised controlled trials, more research is needed in this area. A meta-analysis incorporating recent conflicting randomised controlled trials is urgently warranted.

### Limitations

The main limitation of this integrated review was the quality of studies. The majority of the studies were descriptive and conducted at a single centre with few multicentre randomised controlled trials. A meta-analysis of these studies was not conducted due to the heterogeneity of the interventions, variability in primary endpoints, length of follow-up and study design. In terms of the quality of the evidence, eight of the 17 non-randomised studies were rated as low due to a lack of controlling for potential confounding variables so their results should be interpreted with caution. There were also two studies that had, to date, not published their outcome data so the quality of their evidence was also rated as low. However, it was important to include these studies in the review due to the innovative and potentially effective programs being implemented. Two of the six randomised controlled trials had their risk of bias assessed as unclear. In both of these studies only the abstract was available so full information to assess risk of bias was unable to be accessed.

## Conclusion

A suite of interventions, co-ordinated by a heart failure specialist workforce, are needed across the continuum of care to improve the translation of evidence into practice in patients diagnosed with heart failure. In primary care, collaborations between the general practitioner and cardiologist have been effective at improving evidence-based practice. During hospitalisation, quality improvement programs have improved the quality of inpatient care. In the transitional care phase, heart failure programs, nurse-led clinics, and early outpatient follow-up, reduced hospital readmissions. Importantly, there needs to be a seamless transition of care across the continuum with improved communication and co-ordination between services.

Clinical guidelines recommend evidence-based practice that improves patient outcomes. However, the translation of evidence into practice is lacking. More work needs to be done to bridge the evidence-practice gap to improve outcomes for heart failure patients and to reduce hospital readmissions.

## Appendix 1

**Table 3:** Search strategies

Database searched	Results 31^st^ July 2015
MEDLINE, 1^st^ January 2008 to 31^st^ July 2015	145
CINHAL , 1^st^ January 2008 to 31^st^ July 2015	21
EMBASE, 1^st^ January 2008 to 31^st^ July 2015	107
THE COCHRANE LIBRARY, *Issue* 7 of 12, July (2015). 1^st^ January 2008 to 31^st^ July 2015	9
Grey Literature 1^st^ January 2008 to 31^st^ July 2015	238
Total (including duplicates)	520

1. Medline and Cinahl and Cochrane search strategy

#1 “heart failure” OR “cardiac failure”

#2 prevent* OR reduc* OR improv* OR avoid* OR readmi* OR recommend*

#3 (#1 AND #2)

#4 "care model" OR “model of care” OR "system* of care" OR "care management" OR "patient-cent* care"

#5 (#3 AND #4)

#6 (emergency OR acute OR primary OR community OR after OR ambulatory OR transition* OR aged) N3 care

#7 “acute hospital” OR “general practice”

#8 (#6 OR #7)

#9 (#5 AND #8)

2. EMBASE search strategy

**Table:**

#1	'heart failure'
#2	'cardiac failure'
#3	#1 OR #2
#4	prevent OR prevention
#5	reduce OR reduction
#6	improve OR improvement
#7	avoid OR avoided OR avoidance
#8	readmit OR readmission OR readmissions
#9	recommend OR recommendations
#10	#4 - #9 OR
#11	#3 AND #10
#12	'care model'
#13	‘model of care’
#14	'system of care'
#15	'systems of care'
#16	'care management'
#17	'patient centered care’
#18	‘patient centred care’
#19	#12 - #18 OR
#20	#11 AND #19
#21	(emergency NEAR/3 care)
#22	(acute NEAR/3 care)
#23	(primary NEAR/3 care)
#24	(community NEAR/3 care)
#25	(after NEAR/3 care)
#26	(ambulatory NEAR/3 care)
#27	(transition NEAR/3 care)
#28	(transitional NEAR/3 care)
#29	(aged NEAR/3 care)
#30	‘acute hospital’
#31	‘general practice’
#32	#21 - #31 OR
#33	#20 AND #32

3. Grey literature search strategy

Google Advanced :-

("heart failure" OR "cardiac failure") AND (policy OR guideline) AND (prevent OR prevention OR reduce OR reduction OR readmission OR improve OR improvement) AND ("care model" OR "model of care" OR "management systems" OR "system of care" OR "patient centered care" OR patient centred care”)

- PDF and Microsoft Word document file types only
- .gov and .org domains

## Appendix 2

### Included studies of systems of care for heart failure

**Table 4:** Included studies of systems of care for heart failure (excluding systematic reviews, meta-analyses and policy documents)

Authors, year	Sample	Study design	Intervention	Outcomes
Workforce				
Zuily, 2010 [15]	3200 patients admitted to hospital with ADHF from 1997 to 2007	Pre and post-test design	A heart failure unit was implemented in 2002. All patients received an outpatient appointment with the unit within one month post-discharge. The visits included patient education, assessment with the Cardiologist and up-titration of medications. Patients were followed up monthly with six weekly education sessions.	heart failure related readmissions were reduced from 21.7 % in 2002 to 15.6 % in 2007 (*p* < 0.0001)
Boom, 2012 [13]	7634 patients hospitalized for ADHF who were participating in the EFFECT trial	Retrospective cohort study	Patients were divided as to whether they received cardiologist, general practitioner, or general practitioner with cardiology consultation	Patients treated by general practitioners alone had higher risk of 30-day (OR 1.50, 95 % CI 1.18–1.91) and 1–year mortality (OR 1.29, 95 % CI 1.10–1.50)
NICOR, 2012 [12]	36 504 patients admitted to hospital with heart failure	UK national audit consisting of retrospective review of medical records	Not applicable	In-patient mortality rates: -Cardiology 8 % -Gen Med 14 % Outpatient clinics mortality -Cardiology 18 % -non Cardiology 31 % Community follow-up mortality -Heart failure nurse 22 % -non heart failure nurse 27 %
Comin-Colet, 2014 [16]	2083 patients admitted with ADHF to the hospital with a heart failure service compared to 54 659 patients admitted to hospitals with no heart failure service	Retrospective cohort study	Implemented a health service wide heart failure program encompassing an inpatient service, community service and a heart failure unit including a multidisciplinary specialist heart failure team.	Patients admitted to the hospital with the heart failure service had a lower risk of death (hazard ratio 0.92, 95 % confidence interval, 0.86–0.97), 29 % less likely to experience a readmission for any cause (95 % confidence interval, 0.66–0.76), and 14 % lower risk of heart failure readmissions (95 % confidence interval, 0.80–0.94)
Primary care				
Lee, 2010 [18]	10 599 patients who presented with heart failure and were discharged from an ED in Ontario	Retrospective cohort study	Patients were divided into one of three groups: collaborative follow-up with a cardiologist and primary care physician, primary care physician only follow-up, and no follow-up	Collaborative follow up with a cardiologist and primary care physician reduced 30 day mortality compared to primary care physician only (HR 0.79; 95 % CI 0.63 to 1.00).
Rosstad, 2013 [17]	19 clinicians participated in focus groups.	Qualitative study, focus interviews	27 clinicians were identified as clinical champions to facilitate the implementation of clinical pathways. Focus groups were conducted to discuss the implementation of the care pathway.	A disease-orientated care pathway was not sustainable or appropriate to use in primary care.
In-hospital studies				
Williams, 2010 [21]	Patients admitted to hospital with HFrEF. 50 patients were allocated to the historical group and 47 to the transitional care group	Quasi-experimental design. All patients were followed up for 18 weeks.	In-hospital education and follow-up arrangements either an appointment at the nurse-led clinic or home visits by the community heart failure nurse.	30 day readmissions were lower in the transitional care group at 8 % vs 14 % in the historical group.
Tuso, 2014 [22]	2076 hospital readmissions within 30 days post discharge from a hospitalisation for heart failure	Prospective cohort study	Implemented a heart failure “bundle” that included inpatient heart failure education, a home visit within 48 h of discharge, a follow-up appointment with a physician and a follow-up phone call from a heart failure care manager within 7 days of discharge.	Readmissions rates were reduced from 19 to 15 % over 30-days (*p* = 0.03).
In-hospital clinical audits/registries/quality improvement initiatives				
Boutwell, 2011 [33] (State Action on Avoidable Rehospitalizations initiative- STAAR program)	148 hospitals throughout the US	Quality improvement program.	Hospitals work in partnership with providers and community services that the hospital frequently uses to collaborate in improving communication and coordination during transition from the hospital to the next setting of care.	No results published to date
Heidenreich 2012 [26] (GWTG-HF program)	over 5 million patients and over 2093 US hospitals participate at least once in GWTG throughout the US	Quality improvement program.	Registered hospitals receive access to GWTG toolkit specific for heart failure. The toolkit comprises of: initiation of evidence-based medications, implantation of appropriate device therapies, discharge education about heart failure, evaluation of left ventricular function, and post discharge follow-up appointment. Data is then entered into a web based system and each participating hospital receives site level patient data to identify those at risk of readmission.	Hospitals participating in GWTG-HF had significantly higher documentation of the left ventricular ejection fraction (93.4 % versus non-participating hospitals (89 %), use of ACEI or angiotensin receptor antagonist (88.3 % versus 86.6 %), and discharge instructions (74.9 % versus 70.5 %). After discharge, all-cause readmission at 30 days was 24.5 % and mortality at 30 days after admission was 11.1 %. 30-day readmission was lower for GWTG hospitals (−0.33 %; 95 % CI, −0.53 % to −0.12 %).
Hansen, 2013 [32] BOOST program	11 hospitals throughout the US. In Feb 2014, 180 hospitals were participating. Patient numbers are not mentioned	Quality improvement program. Pre and post implementation design.	The BOOST intervention consisted of a toolkit which contained: an implementation guide, project management tools, such as the Teach Back Training Curriculum, and PICO guidelines to evaluate the intervention; face‐to‐face training and 12 months of expert mentoring and coaching and assistance to build a culture that supports organisational change to reduce hospital readmissions, also linking with other participating sites and data management. Each site also received project benchmark data and site level patient data.	The average rate of 30-day rehospitalization prior to implementation was 15 % and 13 % 12 months later. This was an absolute reduction of 2 % and a relative reduction of 14 %.
H2H National Quality Improvement Initiative, 2015 [34] (H2H program)	No data available	Quality improvement program.	The Hospital to Home (H2H) Initiative provides a toolkit to clinicians assist them in implementing evidence-based care from clinical guidelines, within their organisation.	No data available
Transitional care				
Jaarsma, 2008 [44]	1023 patients were enrolled post hospitalisation for ADHF. Patients were assigned to 1 of 3 groups: a usual care group, a HF nurse follow-up post-discharge and intensive support by a HF nurse.	RCT	The usual care group consisted of follow-up with a Cardiologist within two months post-discharge and then six monthly. Patients in group 2 had nine clinic visits with a HF nurse post-discharge, in addition to the usual care visits. Education about HF and self-management strategies were provided during the HF nurse clinic visits. Group 3 received the same visits as Group 2 and then also received one home visit and weekly telephone contact during the first month post-discharge. After the first month, they also received two additional home visits and two visits with the multidisciplinary team. All patients were follow up for 18 months.	Neither moderate nor intensive follow up by a HF nurse reduced the combined end points of HF death and hospitalization compared with usual care. At 18 months, 411 patients (40 %) were readmitted because of HF or died from any cause: 42 % in the control group, and 41 % and 38 % in the basic and intensive support groups, respectively (hazard ratio, 0.96 and 0.93, respectively; *P* = .73 and *P* = .52, respectively). All-cause mortality occurred in 29 % of patients in the control group, and there was a trend toward lower mortality in the intervention groups combined (*P* = .18).
Driscoll, 2011 [45]	Thirty-three community-based heart failure program coordinators recruited 484 patients diagnosed with systolic dysfunction and >1 earlier hospitalization for ADHF	Cohort study. All patients were followed up for six months.	Patient outcomes in programs with nurse-led titration (NLT) of beta-blockers were compared with those in programs that did not allow such titration.	At 6 months, 47 % of patients participating in UC programs had no change in dosage from baseline to 6 months, compared with 39 % of patients participating in NLT programs. Patients in NLT programs were also more likely to be prescribed at target dose (48 % NLT vs 36 % UC). The composite of all-cause hospitalizations and mortality was lower in patients participating in programs allowing NLT (HR 0.58, 95 % CI 0.42–0.81).
Outpatient clinics				
Fonarow, 2011 [51] IMPROVE program	15,381 patients with HFrEF from 167 US outpatient cardiology practices	Prospective clinical registry	No invention as it was a clinical registry	Adherence to a range of guideline-recommended heart failure therapies ranging from 30–80 %. An increase in adherence in performance measures was significantly associated with improved survival
Driscoll, 2014 [46]	13 patients diagnosed with HFrEF were randomised to usual care and 12 to the NLT clinic	RCT	Patients were randomised to optimisation of BB in a nurse-led titration (NLT) clinic, led by a nurse specialist with the support of a cardiologist in a heart failure clinic, or by their primary care physician	The time to maximum dose was shorter in the NLT group compared to the UC group (90 ± 14 vs 166 ± 8 days, *p* < 0.0005). At six months, in the NLT group there were 82 % on high dose and 9 % on low dose beta-adrenergic receptor blocker compared to the UC group with 42 % patients reaching maximum dose and 42 % patients on low dose.
Fenner, 2014 [50]	Patients admitted to hospital with ADHF.	No data was available.	Patients were seen in hospital and given education by a heart failure nurse. Patients had an appointment scheduled within three days post-discharge to attend the Heart Success Transition Clinic (HSTC). They were seen in the clinic for 4–6 weeks and then referred back to primary care. A telemedicine clinic was also available for patients living in rural and remote areas.	HSTC found a reduction in heart failure readmission rate from 17.92 % to 13.49 %. The telemedicine clinic reduced heart failure readmissions from 18 % to 10 % over a six month period
Telemonitoring programs				
Piette, 2008 [60]	52 heart failure patients and their carers	Prospective cohort study	Telemonitoring using informal carers. The CarePartner Program included an automated telephonic heart failure assessment and behaviour change service. Patients received weekly calls from the system and reported information about their health and self-care using their touchtone telephone. Care nurse managers were notified when a patient reported an urgent medical condition.	75 % of patients had made changes in their self-care as a result of the intervention.
Schwarz, 2008 [59]	102 patients and their carers post discharge from hospital with ADHF	RCT 90 day follow-up	Participants were interviewed within 10 days post discharge and 90 days later. The patient recorded their weight and vital signs daily via the telemonitoring system and responded to questions about symptoms. The data from the telemonitoring system was monitored daily by a heart failure nurse. Usual care consisted of follow-up with their primary physician or cardiologist.	No significant difference in hospital readmissions or mortality
Woodend, 2008 [57]	121 patients admitted with HF and 128 patients admitted with angina	RCT 90 day follow-up	The telemonitoring system consisted of video conferencing and phone transmission of weight, vital signs and ECG. The patient was required to record their weight and vital signs via the telemonitoring system daily and weekly video conferencing with the heart failure nurse. Usual care consisted of follow-up with their primary physician or cardiologist.	No significant difference in hospital readmissions or mortality for heart failure patients
Chaudhry, 2010 [55]	1653 patients admitted with ADHF	RCT	The telemonitoring group was instructed to make daily, toll-free calls to the system. During each call, patients heard a series of questions about general health and heart-failure symptoms. The protocol required the sites to contact any patient whose response generated an alert.	No significant difference in hospital readmissions or mortality
Koehler, 2011 [52]	710 chronic heart failure patients	RCT	The telemonitoring program consisted of: portable devices for ECG, blood pressure, and body weight measurements. Patients were required to undertake daily self-assessments and the data was transferred to the telemonitoring data centre. Usual care consisted of follow-up with their primary physician or cardiologist.	No difference in mortality or heart failure hospitalisations between groups.
Angermann, 2012 [39]	715 patients hospitalised with systolic heart failure	RCT All patients were followed up for 180 days.	The telemonitoring intervention consisted of: inhospital visit, structured telephone follow-up addressing heart failure symptoms, medications, health systems utilisation and psychological well-being; titration of heart failure medication, and increased access to specialist care. Contact was weekly for one month and then individualised based on NYHA class. Usual care consisted of follow-up with their primary physician or cardiologist.	No difference in mortality or hospitalisations between groups.
Dendale, 2012 [54]	160 patients hospitalised with ADHF from seven hospitals	RCT Six month follow up	Patients were asked to transmit their weigh and vital signs daily via the telemonitoring system. When these measurements exceeded preset limits for two consecutive days an automatic email alert was sent to their primary physician and heart failure clinic. Their primary physician was to contact the patient when they received an alert and the heart failure nurse would follow up with the patient 1–3 days post alert. Usual care and telemonitoring patients were all seen in the heart failure clinic 2 weeks post-discharge. Usual care consisted of follow-up with their primary physician post-discharge.	All-cause mortality was significantly lower in the TM group as compared with the UC group (5 % vs. 17.5 %, *P* = 0.01). The number of heart failure readmissions per patient showed a trend (0.24 vs. 0.42 hospitalizations/patient, *P* = 0.06) in favour of TM.
Pekmezaris, 2012 [56]	168 patients hospitalised with a primary or secondary diagnosis of heart failure	RCT All patients were followed up for 90 days.	The telehealth intervention consisted of two video-based nursing visits (including weighs and monitoring of vital signs) and one visit with a community heart failure nurse within the first two weeks post-discharge. The frequency of the telehealth visits was determined the heart failure nurse based on patient needs and continued for 90 days post-discharge. Usual care consisted of follow-up with a community heart failure nurse.	No significant difference in hospital readmissions or mortality
Baker, 2013 [61]	3534 patients with chronic heart failure, chronic obstructive pulmonary disease, or diabetes mellitus. Intervention group (*n* = 1,767) and in the matched control group (*n* = 1,767)	Retrospective matched cohort study. Two years of follow-up	The Health Buddy Program, which integrated a content-driven telehealth system with care management.	The Health Buddy Program had 15 % lower risk-adjusted all-cause mortality (HR 0.85, 95 % CI 0.74–0.98) and reductions in inpatient readmissions during the study period that were 18 % greater than those of matched controls during this same time period (−0.035 vs −0.003; difference-in-differences = −0.032, 95 % CI = −0.054 to −0.010).
Krum, 2013 [53] CHAT study	405 patients diagnosed with heart failure. 217 patients were randomised to usual care by their primary physican and 188 to the intervention group.	Cluster deign trial with randomisation at level of General Practitioner. All patients were followed up for 12 months	The intervention comprised of ongoing support by touchtone telephone using the ‘TeleWatch’ system. Patients were required to dial in monthly to receive advice about the management of their heart failure and to complete education modules about the management of heart failure at home. Patients also had access to heart failure specialist nurse via the Telewatch system.	Fewer patients hospitalised for any cause (74 versus 114, adjusted HR 0.67, 95 % CI 0.50–0.89) and who died or were hospitalised (89 versus 124, adjusted HR 0.70 (95 % CI 0.53–0.92), in the intervention group vs usual care group, respectively.
Black, 2014 [58]	No data available	Multicentre RCT	Patients in the intervention group will receive intensive patient education using the ‘teach-back’ method and receive instruction in using the telemonitoring equipment. Following hospital discharge, they will receive a series of nine scheduled health coaching telephone calls over 6 months from nurses located in a centralized call center. The nurses also will call patients and patients’ physicians in response to alerts generated by the telemonitoring system, based on predetermined parameters.	No published results to date

*ADHF* acute decompensated heart failure, *OR* odds ratio, *HR* hazard ratio, *CI* confidence intervals, *ACEI* angiotensin converting enzyme inhibitors, *HFrEF* heart failure with reduced ejection fraction, *HTM* home telemonitoring, *NTS* nurse telephone support

## Abbreviations

BOOST: Better outcomes for older adults through safe transitions
CHAT: Chronic heart failure assessment by telephone
GWTG: Get with the guidelines
HFpEF: Heart failure with preserved ejection fraction
HFrEF: Heart failure with reduced ejection fraction
HNC: HeartNetCare
HR: Hazard ratio
IMPROVE: Improve the use of evidence-based heart failure therapies in the outpatient setting
NOS: Newcastle-Ottawa Scale
OR: Odds ratio
RCT: Randomised controlled trial
RR: Relative risk
STAAR: State action on avoidable rehospitalisations
TEMA-HF: Telemonitoring in the management of heart failure
